# Structure and Function of Filamin C in the Muscle Z-Disc

**DOI:** 10.3390/ijms21082696

**Published:** 2020-04-13

**Authors:** Zhenfeng Mao, Fumihiko Nakamura

**Affiliations:** School of Pharmaceutical Science and Technology, Tianjin University, Tianjin 300072, China; zhenfengmao@tju.edu.cn

**Keywords:** Filamin C, FLNC, sarcomere, Z-disc, mutation, filaminopathy, myopathy

## Abstract

Filamin C (FLNC) is one of three filamin proteins (Filamin A (FLNA), Filamin B (FLNB), and FLNC) that cross-link actin filaments and interact with numerous binding partners. FLNC consists of a N-terminal actin-binding domain followed by 24 immunoglobulin-like repeats with two intervening calpain-sensitive hinges separating R15 and R16 (hinge 1) and R23 and R24 (hinge-2). The FLNC subunit is dimerized through R24 and calpain cleaves off the dimerization domain to regulate mobility of the FLNC subunit. FLNC is localized in the Z-disc due to the unique insertion of 82 amino acid residues in repeat 20 and necessary for normal Z-disc formation that connect sarcomeres. Since phosphorylation of FLNC by PKC diminishes the calpain sensitivity, assembly, and disassembly of the Z-disc may be regulated by phosphorylation of FLNC. Mutations of FLNC result in cardiomyopathy and muscle weakness. Although this review will focus on the current understanding of FLNC structure and functions in muscle, we will also discuss other filamins because they share high sequence similarity and are better characterized. We will also discuss a possible role of FLNC as a mechanosensor during muscle contraction.

## 1. Introduction

Filamin C (FLNC) protein is one of three filamin isoforms (A, B, C) that cross-link actin filaments (F-actin) and interact with various binding partners [1,2]. Although Filamin A (FLNA) and Filamin B (FLNB) are ubiquitously expressed [3], FLNC is most prevalent in skeletal and cardiac muscles and localizes to Z-discs, myotendinous junctions, the sarcolemma and intercalated discs [4,5,6,7]. However, FLNC is detectable in many tissues and tissue culture cells other than heart and skeletal muscle [4,8]. In this review, we will focus on the structure and functions of FLNC in normal and patho-physiology, but we will also discuss other filamins because they have high homology (>70%) and both common and isoform-distinct functionalities [2,9]. However, expression of FLNA and FLNB are not detected in the Z-disc of developed skeletal and cardiac muscles [7,10,11]. Instead, FLNA and FLNB are concentrated in vascular endothelial cells [10,12], although low expression of FLNB is detected in skeletal muscle (https://www.proteinatlas.org/ENSG00000136068-FLNB/tissue/skeletal+muscle).

The importance of the FLNC gene was highlighted when it was found to be associated with myofibrillar myopathy in skeletal and cardiac muscles [13,14,15,16]. This review will integrate contemporary data based on disease phenotypes, DNA sequencing, and biochemical and cell biological studies of FLNC protein to discuss common and distinct disease mechanisms.

Recent research revealed that FLNA mediates mechano-sensing and mechano-transduction and FLNC may also participate in mechano-transduction [2,17,18,19]. Therefore, this review will discuss how FLNC and FLNA may similarly and differently mediate mechanotransduction.

## 2. Structure of Filamin C (FLNC) and Its Family Proteins

**Figure 1:**
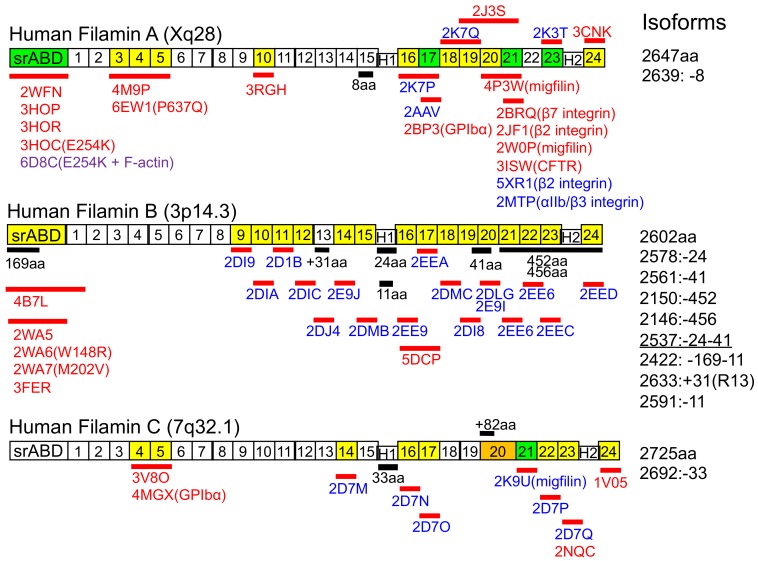
Schematic structures of human filamins (FLNs). All three FLNs consist of an N-terminal spectrin-related actin-binding domain (srABD) followed by 24 Ig-like repeats with two intervening calpain-sensitive hinges. Black bars indicate unique amino acid residues in each isoform. Solved domain structures are indicated in red bars with PDB accession numbers in red (X-ray crystallography), blue (NMR), and purple (cryo-EM). Green indicates a domain whose structure is solved as a complex with a binding partner. Yellow indicates a domain whose structure is solved. srABD, spectrin-related actin-binding domain; H1, hinge-1: H2, hinge-2. Note that the figure indicates the embryonic forms of Filamin C (FLNC) and that hinge-1 of FLNC is spliced out during myogenesis.

The human FLNC gene is encoded in chromosome 7q32.1, comprising ~29.5 kb of genomic DNA and containing 48 coding exons [3,4,5,20,21]. Sequence alignment revealed that FLNC protein is very similar to other filamins (72.8% to FLNA, 71.2% to FLNB) that consist of N-terminal spectrin related actin-binding domain (srABD) followed by 24 immunoglobulin (Ig)-like repeats (R1-24) with two intervening calpain-sensitive hinges separating R15 and R16 (hinge 1) and R23 and R24 (hinge-2) (Figure 1). However, FLNC contains an insertion of 82 amino acids in R20 and hinge-1 is spliced out during myogenesis [5,22]. In fact, the splice variant that is predominantly expressed in skeletal and cardiac muscles lacks exon 32 that encodes the hing-1, resulting in a protein of 2692 amino acids [4]. The hinge-1 of FLNB is also removed during myogenesis but not in FLNA [22]. Although the structure of FLNC strand A is not known (strand A of FLNC R16 in PDB:2D7N is missing), it is likely that FLNC strand A is free from the folded structure because strand A of R16 of FLNA and FLNB are not folded (PDB: 2K7P and 2EE9) and their sequences are similar to that of FLNC. Therefore, some flexibility between R15 and R16 could be retained without hinge-1. Interestingly, the splicing of the hinge-1 is correlated with switching of integrin expression from β1A to β1D during myogenesis in mouse C2C12 myoblast [22].

The splice variant without hinge-1 is also expressed in other tissue such as bone marrow and thyroid, but a biological significance of the splicing is not known [4]. Because purified FLNC protein is not available, biochemical and biophysical properties of full-length FLNC have not been studied. Nevertheless, it is likely that FLNC has similar functions according to the sequence similarity to FLNA and B. Therefore, we briefly describe the structure of FLNA which is better characterized than other filamins. Detailed reviews of FLNA structure are available in other journals [2,18]. The N-terminal srABD consists of two calponin homology domains (CH1 and CH2) that are found in many other actin-binding proteins [23]. Although the atomic structure of FLNC srABD is not available, its sequence similarity to the srABD of FLNA and B is over 90%. Their differences are concentrated in the very N-terminal sequences before CH1 and the linker between CH1 and CH2 [24]. Currently the biological significance of these differences is not known.

**Figure 2:**
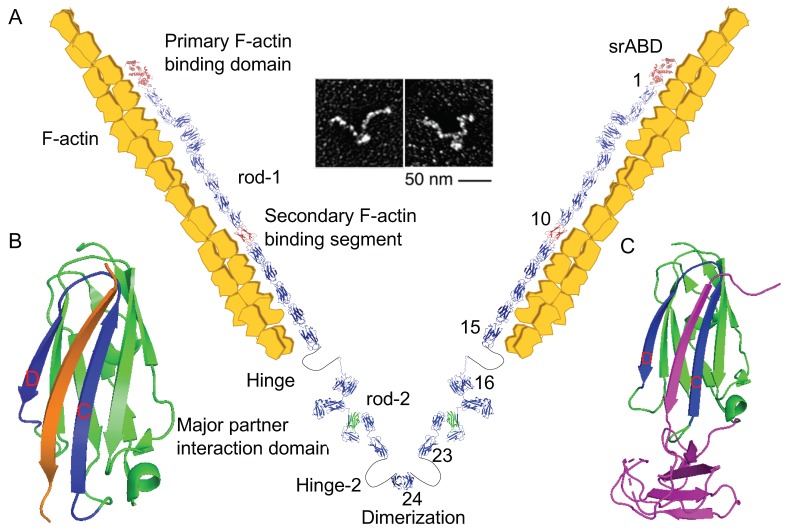
Current atomic structure of Filamin A (FLNA) cross-linking F-actin. (**A**) FLNA is a 560 kDa dimer having spectrin-related actin-binding domain (srABD) followed by 24 Ig-like repeats. Repeats highlighted in red bind along F-actin (yellow). The green labeled domain (R21) is the cryptic integrin binding domain. (**B**) Structure of R21 complexed with cytoplasmic domain of integrin beta7 (orange). All the FLNA-partner complexes that have been structurally revealed use the cleft formed by C and D strands indicated in blue. (**C**) Structure of domain pairs of R20-R21. Note that the CD cleft are blocked by strand A of R20 (magenta).

Atomic structures of the individual Ig repeats have been determined by Nuclear Magnetic Resonance (NMR) analysis and X-ray crystallography as summarized in Figure 1. The Ig domains are composed of seven strands (A through G). Although the repeats were predicted to be linearly aligned to form rods (rod-1, R1-R15; rod-2, R16-R23, Figure 2) based on the V-shaped structure of the purified FLNA molecule [25], structural analysis of the shorter fragments of the rods revealed domain-domain pairs in R3-R5, R16-R17, R18-R19, and R20-R21 [26,27,28,29] (Figure 2). Small-angle X-ray scattering revealed a compact structure in FLNC R3-R5 and possibly in R11-R12 and R14-R15 [29,30]. These domain pairs can be unfolded by mechanical forces to expose cryptic binding site as discussed below (mechanotransduction). Interestingly, all currently reported structures of filamin-partner complexes demonstrated that these binding partners interact through the cleft formed by C and D strands, which is blocked in some domain pairs [2,18] (Figure 2). However, effect of the insertion of 82 amino acids in R20 on the domain pair of R20-R21 is not known. In addition, loss of hinge-1 and insertion of 82 amino acids in R20 of FLNC would influence the structure of full-length FLNC molecule but purified FLNC molecule is currently not available to investigate. Such a unique structure of FLNC, if any, could determine the function of FLNC in muscle Z-disc. 

FLNA possesses the secondary F-actin binding site in rod-1, and R10 alone can decorate purified F-actin [31]. Although the srABD is necessary and sufficient for F-actin binding, the secondary ABD on rod-1 is necessary for high-avidity F-actin binding [27]. The R10 of FLNC shares high sequence similarity with that of FLNA (86% homology), but F-actin binding activity of FLNC R10 has never been examined.

The last repeat, R24, of FLNA is sufficient to form an L-shaped homodimer [27], but biochemical and yeast two hybrid analysis demonstrated that heterodimerization between different filamin isoforms is possible [32,33]. This could be a reason why FLNB occasionally localized at the Z-discs in cultured C2C12 myoblasts by forming a heterodimer with FLNC [22]. It is also possible that dimerization or even oligomerization of filamin subunits are mediated by their binding partners which are dimerized or oligomerized [34].

## 3. FLNC Binding Partners

In 2011, we listed over 90 filamin binding partners including channels, receptors, intracellular signaling molecules, and even transcription factor [2]. In the past ten years, the list of filamin binding partners has been expanded and is still expanding [35,36]. Considering the sequence similarity of filamin isoforms, FLNC likely interacts with most of these numerous binding partners if they are co-expressed, although one binding partners has been shown to only interact with filamin A [37]. Here, we focused on summarizing binding partners that were experimentally demonstrated to interact with FLNC in Table 1 with known functions. Importantly, mutations in many of these binding partners such as myotilin, titin, and BAG3 also leads to myofibrillar myopathies that are associated with disintegration of the Z-disc [38]. Although some of these binding partners interact with all filamin isoforms, others may specifically interact with FLNC. For example, muscle specific myotilin interacts with FLNC through the insertion of 82 amino acid residues in R20, which is missing in FLNA and B [7,39]. Despite numerous lists of the binding partners, atomic structures of many of their complexes with FLNC have remained uncharacterized so far (Figure 1). Not many experiments have been performed regarding the function of their interaction. Because deletion of one of the components for functional analysis most likely does not unveil the specific function of their interaction, more pinpoint intervention is necessary without disturbing other functions.

## 4. Topology of FLNC in the Z-Disc

FLNC localizes to Z-discs, myotendinous junctions, the sarcolemma, and intercalated discs of striated muscle [6]. In the Z-disc, FLNC interacts with the calsarcins (FATZs, myozenins) [40,41,42], myotilin [7] and myopodin [43], while at the sarcolemma FLNC directly binds γ- and δ-sarcoglycans [5] as well as ponsin/CAP [44]. FLNC also interacts with XIRP family proteins (Xin and XIRP2) [45,46]. In adult muscle, XIRPs not only colocalize with FLNC at myotendinous junctions of skeletal muscles and intercalated discs of cardiomyocytes, but also at sites of small injuries and remodeling [47,48] resulting from exercise and eccentric contractions [49,50,51]. Consistent with these results, loss of FLNC in mice causes a severe muscle phenotype that includes defects in embryonic myogenesis resulting in a decreased number of primary fibers, excessive fiber size variation, and a disturbance of sarcomere architecture [52]. 

**Figure 3:**
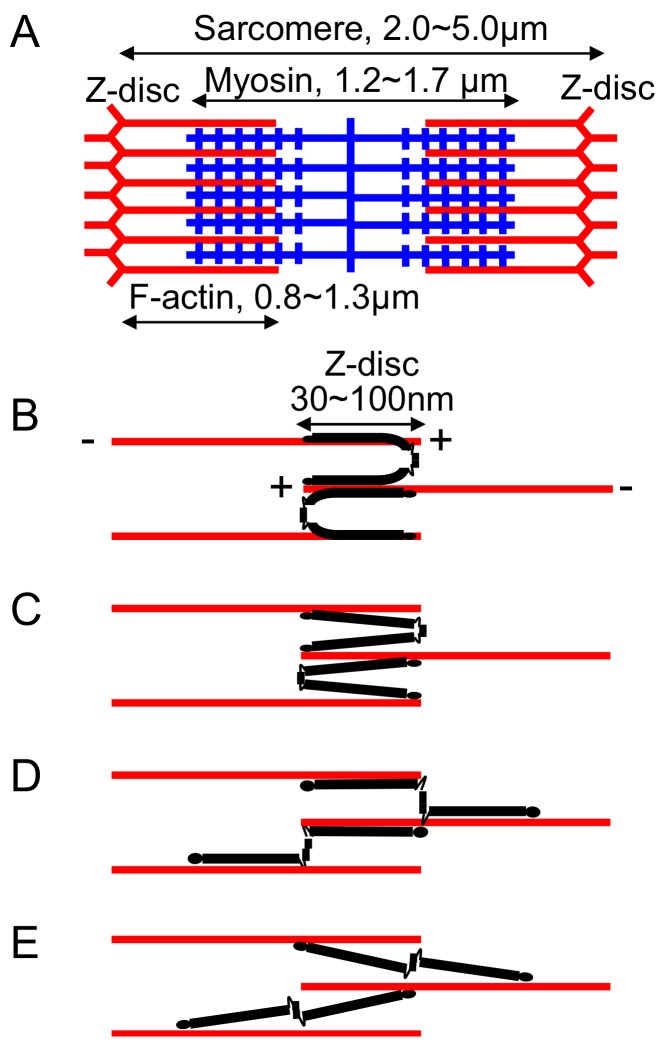
Schematic structure of muscle sarcomere and how FLN connects F-actin in the Z-disc. (**A**) Sarcomere units in striated muscle. The width of the Z-disc: fast fibers, ~30–50 nm; slow and cardiac fibers, ~100 nm. (**B**–**E**) A model of how FLNC (black) cross-links F-actin (red) in the Z-disc. Actin filaments are oriented with their plus ends in the Z-discs and their minus ends toward the center of the sarcomere. Because FLNC also interacts with transmembrane proteins such as integrins and sarcoglycans [58] (Table 1), such connections could also determine topology of FLNC and its binding proteins in the Z-disc. However, since FLNC is highly dynamic in the Z-disc, it is likely that such structure is constantly remodeled which may play a role in the fast repair of myofibrillar microdamage [59].

Ultrastructure of skeletal and cardiac muscle displays a striated sarcomeric pattern with 2.0~5.0μm of sarcomeres which are connected through the Z-disc [53,54,55,56] (Figure 3A). The width of the Z-disc differs in different muscle: fast fibers, ~30–50 nm; slow and cardiac fibers, ~100 nm, being determined by the amount of overlap of F-actin and by the number of Z-link layers ranging from two in fast muscle to six in slow muscle [57]. Histochemical study detects FLNC protein exclusively in the Z-disc despite its binding ability to F-actin that is also expressed in other regions of sarcomere [5,7]. Such specificity is defined by FLNC-binding proteins localized in the Z-disc. As listed in Table 1, several Z-disc specific proteins interact with FLNC. For example, myotilin interacts with FLNC through the unique insertion in R20 and FLNA, which does not contain this insertion, and does not localize in the Z-disc upon overexpression in muscle cells [7]. During muscle development, FLNC mRNA and protein are absent from proliferating cultured human skeletal muscle cells, but up-regulated immediately after the induction of differentiation [6]. Since FLNC localizes in the Z-disc already at the first stages of Z-disc formation, FLNC might play a role in Z-disc assembly. 

Figure 3B–E shows possible models of how FLNC cross-links F-actin in the Z-disc. Actin filaments are oriented with their plus ends in the Z-discs and their minus ends toward the center of the sarcomere. Although FLNA rod-1 domain interacts with F-actin [27,31], it is not known if FLNC rod-1 also interacts with F-actin. If binding of FLNC to F-actin has orientation selectivity, model B and C are not possible. Because the length of FLNA subunit is ~80 nm [27] and FLNC has also 24 repeats, and because both of the two FLNC subunits have binding sites for Z-disc proteins such as titin and myotilin, the width of the Z-disc should be wider in model D and F unless one of the FLNC subunit is located outside of the Z-disc detected by electronmicroscopy. Therefore, it is likely that FLNC connects F-actin in model B or C.

## 5. Association of FLNC Mutations with Myopathies

**Figure 4:**
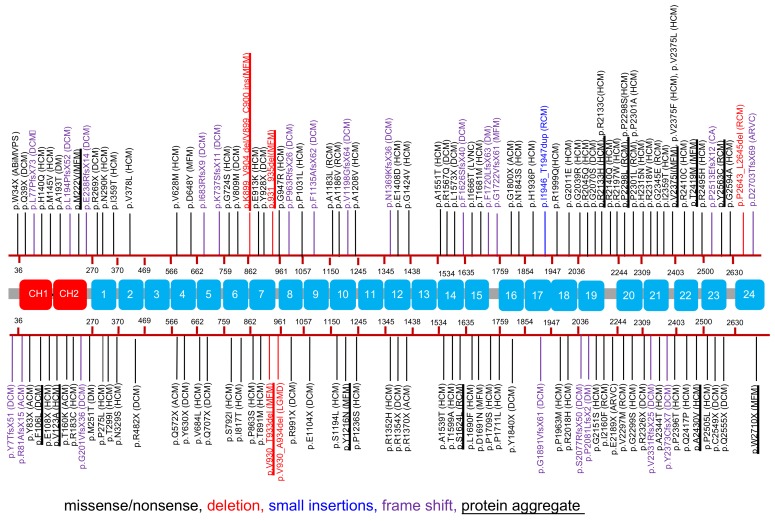
A map of how disease-related FLNC mutations are distributed on FLNC domains. Calponin homology (CH) domains are marked with red; Ig-like domains are numbered from 1 to 24 and marked with light blue. Mutations reported in the literatures are mapped to the protein structure. Mutated positions are labeled in color in accordance with the type of mutations: black, missense/nonsense; blue, small insertions; red, deletion; purple, frame shift. Mutation resulted in protein aggregate is underlined. Assignment of FLNC mutations to the corresponding domain structure was based on the UniProt database. Distal myopathy (DM), Myofibrillar myopathy (MFM), Hypertrophic cardiomyopathy (HCM), Restrictive cardiomyopathy (RCM), Dilated cardiomyopathy (DCM), Arrhythmic cardiomyopathy (ACM), Arrhythmogenic right ventricular cardiomyopathy (ARVC), Cardiac arrhythmias (CA), Arrhythmogenic bileaflet mitral valve prolapse syndrome (ABiMVPS), Left ventricular non compaction (LVNC), Limb-girdle muscular dystrophy (LGMD).

Loss of Flnc gene in mice results in severe defects in myogenesis and myotube structure [52]. In homozygous mice, their skeletal muscles are severely affected, whereas the heart has a normal appearance. However, heterozygous mice are viable and fertile without any abnormalities, demonstrating that neither the low level of wild-type Flnc nor truncated Flnc results in an obvious phenotype [52]. Since human FLNC myofibrillar myopathy slowly progresses, with muscle weakness starting at 24–60 years old, these mutant mice could be analyzed at older age. In humans, a wide variety of myopathy-associated mutations and variants have also demonstrated importance of FLNC in muscle development (Figure 4 and Table 2). 

### 5.1. FLNC Mutation in Distal and Myofibrillar Skeletal Myopathy

FLNC mutations have been associated with distal and myofibrillar skeletal myopathies (MFM) [13], characterized by progressive morphological changes of the muscle fibers beginning in the Z-disc and protein aggregates in the sarcoplasm. Mutations in several genes encoding for Z-disc proteins have also been associated with MFM, including myotilin, ZASP, BAG3, desmin, and αB-crystallin [129,130,131,132,133]. The first FLNC-related myopathy was reported in 2005 showing that a nonsense mutation (c.G8130A, p.W2710Ter) in the FLNC dimerization domain causes a disease in a large German family characterized by muscle weakness and typical myofibrillar myopathy features [127]. A few years later, three deletion mutations in FLNC Ig-like domain 7 have been reported: one harboring an internal 12-nucleotide deletion (c.2997_3008del, p.V930_T933del) [126], the second exhibiting an 18-nucleotide deletion/6 nucleotide insertion (c.2695–2712del/GTTTGT ins, p.K899_V904del/V899_C900ins) [125], and a 15-nucleotide deletion (c.2791_2805del, p.931_935del) [102]. These deletions in the FLNC gene cause protein aggregation, abnormalities in muscle structure, and impairment in muscle fiber function, which leads to muscle weakness. 

A frameshift deletion (c.5161delG, p.Gly1722ValfsTer61) (R15) in exon 30 encoding R15 in a Bulgarian family have reported to lead to haploinsufficiency associated with distal muscle weakness primarily in the upper limbs with lower limb involvement upon disease progression [123]. This phenotype differs from those caused by c.577G4A (p.A193T) and c.752T4C (p.M251T) mutations occurring in the actin-binding domain. These mutations have the distinct involvement of hand muscles and induce aggregates of F-actin in cell due to increased affinity of the mutant FLNC to F-actin although the mutant srABD per se are soluble [124]. Interestingly, the p.A193T mutation is also associated with cerebellar and spinal cord abnormalities [93]. Indeed, other studies have shown an association between FLNC and neuronal diseases [120,123,134]. However, another mutation in the srABD (c.A664G, p.M222V) showed a predominant leg involvement and myofibrillar aggregates [94]. The mutation in R 21 (c.7123G > A, p.V2375I) is also associated with distal myopathy with aggregation and lower motor neuron syndrome [90]. These data suggest that mutations in srABD and rod domains cause overlapping and distinct clinical symptoms and histopathological changes. Therefore, the relationship between genotype and phenotype remains unclear and may be affected by as-yet unknown genetic and environmental causes [110].

### 5.2. FLNC Mutation Development in Cardiomyopathy

Cardiomyopathy (CM) is a rare disorder of the heart muscle and the leading cause of heart transplantation in children [135]. As listed in Table 2, several reports suggested FLNC as a causal gene for CM and about one-third of the FLNC myofibrillar myopathy patients showed cardiac abnormalities [13]. For example, mutations of p. S1624L in R14 and p.I2160F in R20 cause familial restrictive CM (RCM), that leads to disorganization of Z-discs. Affected individuals develop heart failure that requires heart transplantation in some cases of children and adults [116]. A splicing mutation in FLNC: c.2389+1G>A could be responsible for cardiac-restricted dilated cardiomyopathy (DCM) [104]. In addition, FLNC variants have been associated with multiple types of CM such as restrictive cardiomyopathy (RCM) and arrhythmic cardiomyopathy (ACM) in the absence of skeletal muscle pathology [16,88,89,112,113,116,119,121,136]. Moreover, a recent report has described that FLNC truncating variant may serve as a proarrhythmic genetic substrate for arrhythmogenic bileaflet mitral valve prolapse syndrome [91]. However, sequencing of FLNC gene from 540 hypertrophic cardiomyopathy (HCM) patients and 307 healthy controls, revealed that FLNC mutations are not rare, even in healthy controls, with a frequency of about 4%. This is far higher than HCM prevalence (0.2%) in the general population. In addition, FLNC-CM patients rarely have skeletal myopathy, suggesting that the genotype–phenotype relationship of some FLNC mutations might be uncertain and needs to be further evaluated [97].

### 5.3. Genotype–Phenotype Correlations in FLNC Mutation 

The genotype–phenotype relationship has great significance for risk management. Among the 117 published FLNC variants, 41 were truncating variants, 70 were missense variants, and 6 were deletion or insertion without shift of the reading frame (Figure 4, Table 2). Regarding the phenotype, 16 variants were associated with a skeletal myopathy phenotype (4 in distal myopathy, 11 in myofibrillar myopathy, and 1 in Limb-girdle muscular dystrophy) and 101 were associated with cardiomyopathy (29 variants in DCM, 50 in HCM, 11 in RCM, 6 in ACM, 2 in arrhythmogenic right ventricular cardiomyopathy (ARVC), 1 in left ventricular noncompaction (LVNC), 1 in Cardiac arrhythmias (CA), and 1 in Arrhythmogenic bileaflet mitral valve prolapse syndrome (ABiMVPS)) (Figure 4). Interestingly, almost all DCM/ACM are caused by loss of function mutations such as nonsense, frameshift, or splicing as discussed more details below. In contrast, HCM/RCM mutations are mainly associated with missense variants.

Location of the variants on FLNC molecules causes distinct and overlapping phenotypes and many of the relationships between genotype and phenotype remain unclear. However, mapping the distribution of disease-related variants on FLNC domains reveals some genotype–phenotype relationships (Figure 4). For example, among the 50 published variants in HCM patients, 49 were missense variants. Interestingly, a larger proportion of variants locates in the rod-2 domain, especially between R19 and R22, revealing a cluster of missense variants in the HCM phenotype [97,111,112,121]. These data suggest importance of the rod-2 presumably because the unique insertion of 82 amino acids in R20 interacts with the Z-disc proteins and the rod-2 mediates mechanotransduction (see below).

FLNC variants causing DCM, one of the leading causes of heart failure, have the most direct evidence of a genotype–phenotype relationship. Of the 42 previously reported DCM variants, 16 are frameshifts variants, 2 are missense variants, 13 are splice site variants, while 11 are nonsense variants resulting in an early protein truncation. One of two missense variants found was associated with a null variant in a neonatal case of DCM [115]. In this case, the congenital form of the DCM could be because of the cumulative effect of the missense variant associated with the heteroallelic truncating variant in FLNC. Genetic screening of large DCM patient cohorts revealed an association with truncating FLNC variants (stop codons, frameshifts, and splice site variants), strongly suggesting an overlap between mutation mechanisms and patient phenotype [109,112].

In conclusion, the mutations are distributed in all domains of FLNC protein having mutational hotspots in R24 (p.Trp2710Ter) that is found in patients from diverse ethnic origins [13,137]. Biochemical analysis revealed that many of the mutations lead to aggregation of FLNC proteins [99,121,137] (Figure 4). For example, expression of four FLNC mutants (p.V123A, p.A1539T, p.R2133H, and p.A2430V) in cardiac tissue culture cells resulted in the formation of actin aggregates, although FLNC p.A1539T mutant protein itself appears to be soluble [121]. Importantly, the p.Trp2710Ter-induced aggregate blocks autophagy through BAG3 recruitment to the aggregate, suggesting that both BAG3 reduction and autophagy promotion as potential therapies for FLNC p.Trp2710Ter myofibrillar myopathy [14]. Some of the mutations of FLNC might facilitate degradation of the mutant proteins. For example, FLNC c.2997_3008del, R7delVKYT increase susceptibility against protease [138]. In FLNA, V711D, and H743P, mutants are more sensitive to chymotrypsin and disrupt the interaction with protein tyrosine phosphatase PTPN12 [139]. However, effects of the FLNC mutations on partner interaction has never been investigated except for actin [124]. These results indicate that different mutations of FLNC result in diseases of different mechanisms.

## 6. Post-Translational Modifications 

PKCα phosphorylates serine residues in mouse FLNC R13, R20, and hinge-2 [140]. Phosphorylation at S2623 and S2624 of human FLNC, which are located in the vicinity of the main calpain 1 cleavage site (Tyr2625), significantly reduces its susceptibility to cleavage [140]. Since the cleavage of hinge-2 removes R24 dimerization domain, mobility of FLNC subunits increases, suggesting that phosphorylation regulates disassembly of the Z-disc [141].

Klhl31, a transcriptional repressor in MAPK/JNK signaling pathway, interacts with FLNC and promote Ub proteasome system-dependent degradation by ubiquitinating FLNC [62]. FLNC can also be ubiquitinated by the cardiac ubiquitin-proteasome system in Fbxl22-dependent fashion [67]. Disruption of Klhl31 in mice resulted in congenital myopathy and knockdown of Fbxl22 leads to progressive reduction of cardiac contractility, suggesting importance of FLNC degradation in normal muscle development.

## 7. Mechanotransduction

Muscles are the major force producing tissue in vertebrates and cardiac tissues are constantly affected by mechanical forces [142]. The muscle Z-disc is not only important for mechanical stability and force transmission, but also for mechanotransduction [143]. Although the function of FLNC as a molecule that mediates mechanotransduction is not known, FLNA has been shown to sense mechanical force through actin cytoskeleton by converting the force to biochemical signal [17,18,144]. Because FLNC is highly concentrated in the Z-disc and structurally similar to FLNA, it is worth discussing what is known about FLNA as a mechanotransducer. Single molecular analysis revealed that unfolding of FLNA rod-2 can occur by relatively weak forces (~10 pN), which is smaller than rupture forces of FLNA/F-actin interaction (40-80 pN) and actin–actin intermolecular force (~110 pN), whereas unfolding of FLNA rod-1 starts to occur at 50 pN [145,146,147,148,149,150]. Since a single myosin head can generate 1.7~4 pN force, a couple of myosin molecules are enough to unfold the rod-2 [151,152]. These data indicate that unfolding of Ig domain requires over 50pN force, whereas dissociation of domain pairs occurs by physiologically relevant force. FLNA R3-R5 in the rod-1 forms domain pairs but dissociation of these domain pairs may require over 50pN or may be below the measurement limit [29,147,153]. The mechanical properties of the FLNC molecule has never been investigated. Because the hinge-1 is spliced out during myogenesis and FLNC has 82 amino acids insertion in R20, the mechanical property of FLNC could be unique. In addition, how the binding partners interact with FLNC molecules in the Z-disc would influence how forces are transmitted to FLNC molecules. For example, if the binding partner(s) intermolecularly connects FLNC subunits (e.g. rod-1), the rest of the C-terminal domain (e.g. rod-2) may not be unfolded because force may not be transmitted to the C-terminal domain.

**Figure 5:**
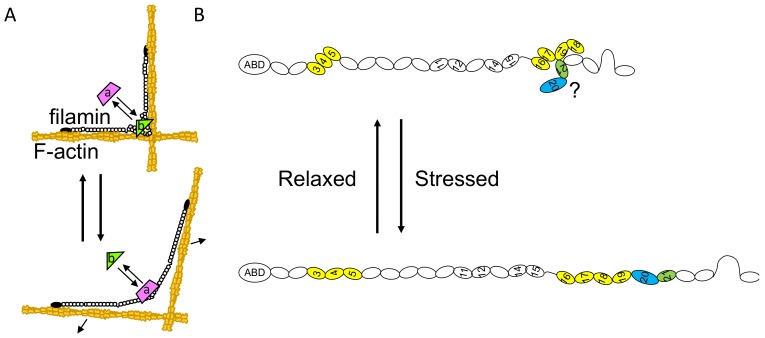
A model of how mechanical forces regulate FLNC-partner interaction. (**A**) Contractile force of actomyosin or deformation of actin networks induces conformational changes of filamin molecule. Some binding partner (a, e.g., beta-integrin) interacts with exposed binding site under mechanical stress, whereas some (b, e.g., FilGAP for FLNA. FilGAP does not interact with FLNC [37]) dissociates when filamin molecule is deformed. (**B**) Predicted structure of FLNC subunit. Only one subunit is shown. The hinge-1 is spliced out during myogenesis but strand A of R16 is free from the folded domain, thereby the link between R15 and R16 possesses some flexibility. Colored domain pairs can potentially be dissociated by mechanical force. Because of unique insertion of 82 amino acids in R20 (blue), it is not known if R20 pairs with R21(green) to create cryptic binding site (Figure 2C). Domain pairs of R11-R12 and R14-R15 were suggested by small-angle X-ray scattering analysis but appears to be loose.

Reconstituted FLNA/F-actin/myosin networks revealed that contraction of actomyosin decreases mobility of beta-integrin that binds to the cryptic binding site in FLNA R21 and increases mobility of FilGAP that binds to R23. This demonstrated that the FLNA molecule is a mechano-sensor and -transducer [17] (Figure 5A). Single molecular analysis also showed force-dependent interaction of R21 with the binding partner [144]. The FLNA/beta-integrin interaction is regulated by opening and closing of the CD cleft of R21, where beta-integrin binds (Figure 2B), whereas the FLNA/FilGAP interaction is regulated by changing the distance between the two R23. However, FLNC does not interact with FilGAP, such a mechanism is not involved in FLNC-partner interaction or FLNC interacts with another binding partner in the similar fashion as FilGAP [37]. Based on the structural analysis of FLNC domains as shown in Figure 1 and small-angle X-ray scattering analysis of the FLNC rod-1 [30], we illustrated a model of FLNC structure in Figure 5B. The structure of FLNC R20 has not been solved and whether or not the CD cleft of FLNC R21 is blocked by domain pair is not known. Nevertheless, due to the sequence similarity of FLNC with FLNA, FLNC may mediate mechanotransduction as well.

## 8. Conclusions and Future Perspective

The ultimate goal of FLNC research should be a cure for FLNC-related myopathy. Although editing the FLNC gene is obviously the best means, preventing the aggregation in the muscle of myopathy patients is another promising strategy. As we unveil the molecular mechanisms of FLNC synthesis and degradation, and the FLNC-mediated signaling pathway, more options should be possible. Revealing how the FLNC molecule is assembled and disassembled in muscle tissue is also beneficial to discovering how to bypass or support mutant FLNC.

## Figures and Tables

**Table 1:** FLNC binding partners.

Binding Partner	Binding Domain on FLNC	Function	Reference
HSPB1(HSP27)	R18-21	HspB1, an abundant molecular chaperone and FLNC form a complex. Phosphorylation of HspB1 facilitates extension of FLNC being localized to load-bearing sites.	[60]
MEK1/2 ERK1/2	Co-IP	FLNC enhances the mitogen-activated protein kinase signaling pathway during tumorigenesis.	[61]
Klhl31	Co-IP	Klhl31 targets Flnc for ubiquitination and degradation.	[62]
HSPB7	R24	Aggregation and mislocalization of FLNC occur in the muscle by loss of HspB7, leading to myopathy.	[63]
KCNE2	Y2H, Co-IP	FLNC and KCNE2, potassium voltage-gated channel, co-localized within the cell, however, a physical interaction was only observed under hypoxic conditions.	[64]
α2C-adrenoceptors	1979 and 2206 (R18-R20) In silico modeling	Phylogenetic and sequence analysis showed that these interactions have evolved in warm-blooded animals.	[65]
Aciculin	Co-IP, SPR (R18-21)	Dystrophin-binding protein aciculin interacts FLNC and Xin in Z-line.	[66]
Fbxl22	Co-IP	FLNC is ubiquitinated in Fbxl22-dependent fashion.	[67]
Ankyrins-G	R5-6	Ankyrins-G contains the muscle-specific Obscurin/Titin-Binding-related Domain that binds to FLNC and plectin.	[68]
Myopodin (synaptopodin2)	R20-21	Myopodin also interacts with other Z-line proteins such as alpha-actinin and zyxin. The interaction might play a role in early assembly and stabilization of the Z-disc.	[43]
IGFN1	R19-24 (Y2H)	FLNC interacts IGFN1 and KY at Z-line	[69]
MKK4 MKK7	Co-IP	MKK4 and MKK7 bind all FLNs. FLNA enhances the activation of MKK7 and JNK.	[70]
BAG3	Co-localization	BAG-3 stimulates the release of filamin from a cytoskeleton. Released filamin could subsequently be ubiquitylated by the CHIP/UbcH5 conjugation machinery in the presence of the E1 ubiquitin-activating enzyme. FLNC^W2710X^ blocks BAG3 mediated clearance of protein aggregates.	[14,71]
CAP (SORBS1, Ponsin)	R2	Cbl-associated protein (CAP) is enriched in oxidative muscle fiber. When overexpressed, CAP recruits FLNC to cell-extracellular matrix adhesions and inhibits FLNC-induced cell spreading on fibronectin.	[44]
USP25m	Y2H	The ubiquitin-specific protease USP25 interacts with three sarcomeric proteins.	[72]
Titin	R20-R24 (Y2H)	Titin Z2-Zis1 domain interacts FLNA/C, alpha-actinin, and nabulin.	[73]
Calpain 1	R23-R24	Calpain 1 cleaves FLNC hinge-2. Phosphorylation of FLNC by PKC alpha protects the proteolysis of FLNC by calpain 1.	[74]
Xin (XIRP1, 2)	R20 (Y2H)	Xin isoforms associate differentially with FLNC. XinB and FLNC compete for binding to aciculin and no ternary complex is formed.	[45,66]
β-arrestin2	R22 (Y2H)	The interaction might regulate dopamine D3 receptor signaling.	[75]
RasGAP	R15-R17	Disrupting the RasGAP-filamin pathway results in reduced myocyte growth.	[76]
Integrin beta1A	R20 (Y2H)		[39]
PKBalpha	substrate	PKBalpha phosphoarylate FLNC Ser2213, which lies in an insert not present in the FLNA and FLNB isoforms. Insulin also induced the phosphorylation of FLNC at Ser2213 in cardiac muscle in vivo	[77]
KY protein	R20-R22 (Y2H)	KY protein cleaves FLNC. Mutation of KY protein disrupts normal distribution of FLNC.	[78]
alpha1-adrenergic receptor	Y2H	Biological significance of the interaction is not known.	[79]
Calpain 3	substrate	FLNC after C3 cleavage, abolishes this interaction with the sarcoglycans.	[80]
N-RAP	R20-24 (Y2H)	During myofibril assembly in cultured chick cardiomyocytes, N-RAP, and filamin appear to co-localize with alpha-actinin in the earliest myofibril precursors found near the cell periphery, as well as in the nascent myofibrils that form as these structures fuse laterally.	[81]
FLNB	R24	Heterodimer formation through R24 is possible between FLNC and B but not between FLNA and the other two filamins.	[33]
LL5beta	Co-IP	LL5beta binds PI(3,4,5)P3	[82]
PKCalpha	R23-24 (Y2H)	Phosphorylates filamins	[83]
Migfilin	R21	Migfilin interacts with Mig-2 and filamin at cell-matrix adhesion site and regulate cell shape change.	[84,85]
SHIP-2 (INPPL1)	R22-23 (Y2H and Co-IP)	Filamin-dependent SHIP-2 localization critically regulates phosphatidylinositol 3 kinase signaling to the actin cytoskeleton.	[86]
Myozenin-1, 2, 3 (FATZ, Calsarcins)	R19-24 (Y2H)	Myozenin interacts with FLNC and alpha-actinin in skeletal muscle Z line.	[39,40,41,42]
KCND2	R20-24 (Y2H)	Filamin is required for Kv4.2 localize at filopodial roots.	[87]
Myotilin	R20 (Y2H)	Insertion of 82 amino acid residue in R20 defines specific localization of FLNC at Z-line and this domain interacts with myotilin.	[7]
γ-, δ-Sarcoglycans	R20-R24 (Y2H)	The identification of FLNC isoform in muscle.	[5,39]
Actin	Predicted from sequence similarity and localization in cells.	High homology to actin-binding domains of FLNA and B.	[5]

Co-IP, Co-immunoprecipitation; Y2H, yeast two hybrid; SPR, Surface plasmon resonance; SPPBA, Solid-phase protein-binding assays.

**Table 2:** Mutations of human FLNC gene.

Mutation/Variant	Phenotype	Reference
c.6565 G>T, p.Glu2189Ter (R20) c.8107delG, p.Asp2703ThrfsTer69 (R24)	Arrhythmogenic right ventricular cardiomyopathy	[88]
7 novel and 2 rare variants	Arrhythmogenic cardiomyopathy	[16,89]
heterozygous missense mutation (c.7123G > A, p.V2375I) in R21	Myofibrillar myopathies with lower motor neuron syndrome	[90]
c.201G>A, p.Trp34Ter (srABD)	Arrhythmogenic bileaflet mitral valve prolapse syndrome	[91]
c.6902C.T, p.Pro2301Leu (R20)	Familial Restrictive Cardiomyopathy	[92]
c.577G > A, p.Ala193Thr (srABD)	Distal and proximal myofibrillar myopathy, cerebellar and CNS sensory ataxia, and pyramidal signs as a consequence of cerebellar and spinal cord abnormalities	[93]
c.A664G:p.Met222Val (srABD)	Distal myofibrillar myopathy	[94]
p.Asp1691Asn (R15) and p.Asp648Tyr (R4)	myopathy	[95]
28 variants, See the reference	Hypertrophic cardiomyopathies, restrictive cardiomyopathies, dilated cardiomyopathy, left ventricle cardiomyopathy	[96]
43 variants See the reference	Hypertrophic cardiomyopathy	[97]
p.Phe1626SerfsTer40 (R14)	Dilated cardiomyopathy with sudden cardiac death	[98]
p.Pro2298Leu (R20) p.Tyr2563Cys (R23)	Restrictive cardiomyopathy	[99]
c.7536_7548del, p.Pro2513GlufsTer12 (R23)	Cardiac arrhythmias	[100]
6 variants See the reference	Arrhythmogenic dilated cardiomyopathy	[101]
c.2791_2805del, p.931_935del (R7)	Myofibrillar myopathies	[102]
c.3557C>T, p.Ala1186Val (R10) c.[3547G>C; 3548C>T], p.Ala1183Leu (R10)	Restrictive cardiomyopathy	[103]
c.2389+1G>A (exon 15 skipping, stop in R6)	Familial dilated cardiomyopathy	[104]
c.6889 G>A, Val2297Met (R20)	Familial Restrictive Cardiomyopathy	[105,106]
c.5161delG, p.Gly1722ValfsTer61 (R15)	Distal muscular dystrophy	[107]
p.Gly2345Glu (R21)	Congenital heart disease	[108]
10 variants	Dilated cardiomyopathy	[109]
c.577G>A, p.Ala193Thr (srABD)	Distal myopathy	[110]
38 variants	Hypertrophic Cardiomyopathy	[111]
23 truncating mutations	Dilated and Arrhythmogenic Cardiomyopathy	[112]
c.7251+1 G>A c.5669-1delG	Dilated cardiomyopathy	[113]
c.3646T>A, p.Tyr1216Asn (R10)	Myofibrillar myopathy	[114]
c.318C>G, p.Phe106Leu (srABD) c.2971C>T, p.Arg991Ter (R8)	Dilated cardiomyopathy	[115]
c.4871C>T, p.S1624L (R14) c.6478A>T, p.I2160F (R20)	Familial Restrictive Cardiomyopathy	[116]
c.2786-2800del, p.V930-A934del (R7)	Limb-girdle muscular dystrophy	[117]
c.969 + 3 A > G	Muscular dystrophy, Congenital myopathy	[118]
c.3791 - 1 G>C	Dilated cardiomyopathy	[119]
p.V831I (R6) Additional 20 variants	Pick’s disease Frontotemporal dementia	[120]
c.4824G>A, p.A1539T (R14) 7 additional mutations	Familial hypertrophic cardiomyopathy	[121]
c.7256C>T, p.Thr2419Met (R22)	Myofibrillar myopathy with late-onset cerebellar ataxia	[122]
c.5160delC, p.Phe1720LeufsTer63 (R15)	Distal myopathy with upper limb predominance	[123]
c.577G>A, p.Ala193Thr (srABD) c.752T>C, p.Met251Thr (srABD)	Distal myopathy	[124]
c. 2695-2712 del/GTTTGT ins, p. Lys899-Val904 del, Val899-Cys900 ins (R7)	Myofibrillar myopathy	[125]
c.2997–3008del, p.Val930_Thr933del (R7)	Myofibrillar myopathy	[126]
c.8130G >A, p.Trp2710Ter (R24)	Myofibrillar myopathy	[14,127,128]

## Abbreviations

FLNC	Filamin C
F-actin	Actin filament
ABD	Actin-binding domain
srABD	Spectrin-related actin-binding domain
CH	Calponin homology
MFM	Myofibrillar myopathy
HCM	Hypertrophic cardiomyopathy
DM	Distal myopathy
RCM	Restrictive cardiomyopathy
DCM	Dilated cardiomyopathy
ACM	Arrhythmic cardiomyopathy
ARVC	Arrhythmogenic right ventricular cardiomyopathy
LGMD	Limb-girdle muscular dystrophy Cardiac arrhythmias
CA	Cardiac arrhythmias
ABiMVPS	Arrhythmogenic bileaflet mitral valve prolapse syndrome
LVNC	Left ventricular non compaction
